# Community Mobility and Depressive Symptoms During the COVID-19 Pandemic in the United States

**DOI:** 10.1001/jamanetworkopen.2023.34945

**Published:** 2023-09-27

**Authors:** Roy H. Perlis, Kristin Lunz Trujillo, Alauna Safarpour, Alexi Quintana, Matthew D. Simonson, Jasper Perlis, Mauricio Santillana, Katherine Ognyanova, Matthew A. Baum, James N. Druckman, David Lazer

**Affiliations:** 1 Massachusetts General Hospital, Boston; 2 Harvard Medical School, Boston, Massachusetts; 3 Northeastern University, Boston, Massachusetts; 4 Rutgers University, New Brunswick, New Jersey; 5 Northwestern University, Evanston, Illinois; 6 Harvard University, Cambridge, Massachusetts; 7 University of Pennsylvania, Philadelphia

## Abstract

**Importance:**

Marked elevation in levels of depressive symptoms compared with historical norms have been described during the COVID-19 pandemic, and understanding the extent to which these are associated with diminished in-person social interaction could inform public health planning for future pandemics or other disasters.

**Objective:**

To describe the association between living in a US county with diminished mobility during the COVID-19 pandemic and self-reported depressive symptoms, while accounting for potential local and state-level confounding factors.

**Design, Setting, and Participants:**

This survey study used 18 waves of a nonprobability internet survey conducted in the United States between May 2020 and April 2022. Participants included respondents who were 18 years and older and lived in 1 of the 50 US states or Washington DC.

**Main Outcome and Measure:**

Depressive symptoms measured by the Patient Health Questionnaire-9 (PHQ-9); county-level community mobility estimates from mobile apps; COVID-19 policies at the US state level from the Oxford stringency index.

**Results:**

The 192 271 survey respondents had a mean (SD) of age 43.1 (16.5) years, and 768 (0.4%) were American Indian or Alaska Native individuals, 11 448 (6.0%) were Asian individuals, 20 277 (10.5%) were Black individuals, 15 036 (7.8%) were Hispanic individuals, 1975 (1.0%) were Pacific Islander individuals, 138 702 (72.1%) were White individuals, and 4065 (2.1%) were individuals of another race. Additionally, 126 381 respondents (65.7%) identified as female and 65 890 (34.3%) as male. Mean (SD) depression severity by PHQ-9 was 7.2 (6.8). In a mixed-effects linear regression model, the mean county-level proportion of individuals not leaving home was associated with a greater level of depression symptoms (β, 2.58; 95% CI, 1.57-3.58) after adjustment for individual sociodemographic features. Results were similar after the inclusion in regression models of local COVID-19 activity, weather, and county-level economic features, and persisted after widespread availability of COVID-19 vaccination. They were attenuated by the inclusion of state-level pandemic restrictions. Two restrictions, mandatory mask-wearing in public (β, 0.23; 95% CI, 0.15-0.30) and policies cancelling public events (β, 0.37; 95% CI, 0.22-0.51), demonstrated modest independent associations with depressive symptom severity.

**Conclusions and Relevance:**

In this study, depressive symptoms were greater in locales and times with diminished community mobility. Strategies to understand the potential public health consequences of pandemic responses are needed.

## Key Points

**Question:**

Were US adults living in communities with decreased mobility during the COVID-19 pandemic more likely to report a depressed mood?

**Findings:**

In this survey study with 192 271 respondents, individuals who lived in communities where fewer individuals left home on a daily basis during the pandemic reported greater levels of depression on average. These differences were not directly attributable to COVID-19 pandemic restrictions, nor to county-level differences in COVID-19 cases or deaths, weather, or county-level economic features; the association persisted after widespread COVID-19 vaccine availability.

**Meaning:**

These findings suggest that the association of depression and policies that restrict social interaction and movement requires further study to understand potential causal relationships and how to mitigate them.

## Teaser

This survey study examines the association of depression and limited mobility during the COVID-19 pandemic in the United States using an internet-based survey of adults in all 50 US states and Washington DC.

## Introduction

Levels of depressive symptoms in the United States have been elevated since early in the COVID-19 pandemic. Multiple large-scale surveys have suggested the prevalence of moderate depressive symptoms up to 3-fold greater than prior baseline estimates,^1,2,3^ with even more conservative analyses finding a 30% to 50% increase in risk for syndromal depression and anxiety.^4^ Although deaths from suicide among adults did not appear to increase, at least in the initial period of the pandemic,^5^ overdose rates increased markedly as well.^6,7^ While the extent to which these symptoms represent major depressive episodes has been debated,^8^ they are difficult to dismiss as a manifestation of overall distress.

An increase in depression in the setting of a massive collective stressor is not surprising. Similar responses have been observed following natural disasters^9^ and wars.^10^ In the setting of the COVID-19 pandemic, there are numerous potential contributors—losses of family members; financial stressors and loss of employment or housing; increased childcare responsibility; consequences of the illness itself; and hopelessness arising from the persistence of the pandemic.^11^ Another stressor, which is not well understood, may be due to the social impact of the pandemic, arising from closures and restrictions required to control the spread of COVID-19.^16^ A study of older adults through July 2020 found an association between stay-at-home orders or restaurant closures and anxiety and depression among older adults,^12^ in accordance with evidence of loneliness in this group.^13^ A broader literature likewise supports the impact of loneliness in this population.^14^ Consistent with exacerbation associated with restrictions, the easing of containment in the UK has been associated with corresponding improvement in multiple measures of mental health in 1 study.^15^

In line with a recent US Surgeon General report on the impact of loneliness and isolation,^16^ we sought to understand whether individuals residing in communities with diminished social interaction experienced greater levels of depression, using data from a large cohort of adults in the United States reflective of national population distributions of age, gender, race and ethnicity, and geography. We elected to focus on community mobility, rather than participant mobility, as this measure is exogenous to the individual (ie, diminishes the potential for reverse causation in which depressive symptoms are manifest in part as diminished mobility). That is, we examined whether the extent to which individuals in a community left home, on average, was associated with levels of depression reported by adults residing in that community. The variation in policies and pandemic severity over time as well as by state provided an opportunity to study the effects of social restrictions while accounting for some of the many potential confounding effects not addressed in prior work.

## Method

### Study Design

This survey study followed the American Association for Public Opinion Research (AAPOR) reporting guidelines. The study design was determined to be exempt by the institutional review boards of Harvard University and Northeastern University; survey participants signed written informed consent online prior to survey access.

We used data from a multiuniversity consortium, the COVID States Project, that has surveyed approximately 20 000 United States adults approximately every 8 weeks since May 2020 on a range of topics related to COVID-19 attitudes and behaviors. The surveys apply representative quotas and nonprobability sampling^17^ to approximate the state-level distribution of age, gender, and race and ethnicity for each of the 50 states and the District of Columbia. Nonprobability sampling represents a lower-cost approach to large-scale sampling that has been shown to yield valid estimates in similar contexts^18,19^; caveats are included in Boyd et al.^20^ The commercial vendor distributed the surveys aggregates across multiple online panels, and members of those panels can opt in to a survey in exchange for modest compensation set by the vendor.

For the present study, we used data from the 18 waves conducted between May 2020 and April 2022. For participants participating in more than 1 wave, in the primary analysis we considered only the index survey in this period; in sensitivity analyses, we considered a random survey or inclusion of all observations with standard errors clustered by individual. All sociodemographic variables were collected by self-report (eMaterials in Supplement 1). The survey asked the respondents to provide their gender using the terms male or female. Respondents were asked to indicate race and ethnicity from a list of options, drawn from standard United States Census categories to allow us to confirm representativeness of the US population by comparing distributions of respondents to known distributions by state. The list included other followed by a free text field for respondents who did not identify with the available choices. Prior COVID-19 positivity was determined by asking, “Have you been tested for coronavirus (COVID-19)?” followed by asking for test result if the answer was yes. The party affiliation variable used in exploratory analysis was collected by asking, “Generally speaking, do you think of yourself as a...”, with follow-up questions to assess strength of affiliation yielding a 7-point ordinal score where 4 is independent.

### Outcomes

Survey participants completed the Patient Health Questionnaire-9 (PHQ-9), a validated screen for major depressive symptoms in outpatient settings that asks about symptom frequency in the prior 2 weeks.^21^ A PHQ-9 score of 10 or greater (out of 0 to 27) identifies moderate or greater major depression with a sensitivity and specificity of 88%^21^ in the outpatient setting, as confirmed by meta-analysis.^22^

### Exposure and Potential Confounding Variables

We quantified human mobility on a given day on a county-by-county basis using Meta (formerly Facebook) movement data.^23^ In brief, these measures of mobility were derived from the mobile application, where users provided permission to track their location. Primary analysis used the metric that captures the proportion of individuals sampled who were only observed in a single Bing tile^24^ (approximately 600 m by 600 m at the equator) on a given day. These would be expected to represent individuals who stayed at home, or very close to home, all day. That is, on this measure, 0 would indicate all individuals left home at least once on a given day and 1 would indicate that none left home. The derivation of this metric and additional considerations are summarized elsewhere.^25^ We calculated 14-day moving averages to correspond to the period assessed by the PHQ-9 (ie, the mean of this measure over the prior 14 days in a given county).

We measured COVID-19 activity, in terms of reported COVID-19 cases and deaths, via the *New York Times*’ curated 14-day rolling average.^26^ For all analyses, both measures were included at the county and state level, recognizing that individuals might be more aware of different levels of acuity (ie, might not be aware of county-level activity). Of note, as New York City results are only reported in aggregate, we assumed each county’s Federal Information Processing System (FIPS) code to have the characteristic of the city as a whole. Other caveats in the collection and curation of these data are described on the *New York Times’* website.^26^

To assess containment policies, we accessed a curated index of daily state-level COVID-19–related restrictions from the University of Oxford’s pandemic response tracking website.^27^ While some city and local governments imposed more stringent restrictions, there is no comprehensive database that captures restrictions below the state-level across the United States. The data include manually curated measures of policy stringency, including school, workplace, and transportation closures, cancellation of public events, restrictions on gatherings, stay-at-home orders, restrictions on internal (within-country) movement, and restrictions on international travel. Based on visual inspection of distributions, in light of their being ordinal but highly nonnormal and in order to maximize interpretability, each individual score was dichotomized prior to analysis to reflect the presence or absence of a particular form of restriction. Specifically, we examined whether or not masks were required in public places; whether at least some school closures were required; whether changes in workplace, such as remote work, were advised; whether individuals were advised to stay at home; whether public transport was reduced or cancelled; whether large gatherings were restricted; and whether public events were cancelled. For purposes of analysis, we calculated the rolling average for the 14 days preceding a given date. For example, a value of 0.5 would indicate that a policy was in effect for 50% of the preceding 14 days.

Because COVID-19 exhibits seasonal patterns and weather can affect individuals’ willingness to go outside, we also examined county-level weather monthly variables drawn from the US National Oceanic and Atmospheric Administration (NOAA).^28^ As summaries are available from NOAA at the county level only on a monthly basis, we derived our own measures using NOAA weather station data via Climate Data Online.^29^ Specifically, for each date we assigned the maximum and minimum temperature (in Fahrenheit degrees relative to 65), and the amount of precipitation (in tenths of mm), recorded at the nearest weather station to the county’s centroid. We then calculated a rolling average of the prior 14 days for each measure. As a proxy for amount of sunlight, we also included latitude of the county centroid in all models.

To address additional potential confounding variables at the county level, we used selected data aggregated in the Atlas of Rural and Small Town America, developed by the Economic Research Service of the US Department of Agriculture.^30^ From the atlas, we accessed 2020 population density based on US Census data and land area, unemployment rate, proportion of adults employed in agriculture and services, poverty and deep poverty rate, average household size, proportion who own their own home, and median household income.

### Statistical Analysis

We used mixed-effects linear regression models to examine associations between county measures of the proportion of individuals remaining at home and total depression severity, as measured by PHQ-9, allowing random intercepts for state. Primary models adjusted for individual participant sociodemographic features including age, gender, race and ethnicity, employment status, household income, education, and number of individuals living in a household. These models also included history of a positive COVID-19 test by self-report, in light of prior work indicating an association between COVID-19 and mood, both during and following acute infection.

We then examined the association of extending these models to incorporate potential confounding variables. First, we added measures of disease activity (in terms of new cases and deaths) at the county, state, and national levels. Second, we added measures of containment to these models to determine whether policies might account for main outcomes. Third, we included climatologic variables, including prior 14-day precipitation, minimum and maximum temperature, and latitude.

Follow-up analyses considered additional county-level measures reflecting population density, employment, and poverty, as described under measures, by adding these variables to the sociodemographic models to assess their role as potential confounders. An additional exploratory analysis added self-reported political affiliation, on a 7-point scale, to regression models.

The stay-at-home measure is an absolute, not relative, measure. To examine the association of the change in the proportion of staying at home, we completed 2 additional sets of analyses. First, we refit the sociodemographic regression model examining 30-day change in mobility score—ie, to what extent did the proportion staying at home increase or decrease compared with 30 days prior. Second, we examined the subset of participants who returned for a second survey, fitting regression models for change in depression severity as the dependent variable and change in the proportion staying at home in their community as the primary independent variable and including the same sociodemographic variables as in all other models.

Mixed-effects models used the lme4 and lmerTest package in R version 4.3.0 (R Project for Statistical Computing), fitting random effects at the state level unless otherwise specified. A nominal 2-tailed *P* < .05 (calculated via Satterthwaite degrees of freedom) was considered to be statistically significant.

## Results

The 192 271 survey respondents had a mean (SD) of age 43.1 (16.5) years; 768 (0.4%) were American Indian or Alaska Native individuals, 11 448 (6.0%) were Asian individuals, 20 277 (10.5%) were Black individuals, 15 036 (7.8%) were Hispanic individuals, 1975 (1.0%) were Pacific Islander individuals, 138 702 (72.1%) were White individuals, and 4065 (2.1%) were individuals of another race. Additionally, 126 381 (65.7%) identified their gender as female and 65 890 (34.3%) as male. Mean (SD) depression severity by PHQ-9 was 7.2 (6.8). Additional characteristics of the cohort as a whole are summarized in Table 1 and additional participant-level features used in regression models are included in Table 2.

**Table 1.** Characteristics of the Study Cohort

Characteristics	Overall, No. (%) (N = 192 271)
Age, mean (SD) y	43.1 (16.5)
Gender	
Female	126 381 (65.7)
Male	65 890 (34.3)
Race and ethnicity	
African American or Black	20 277 (10.5)
Asian	11 448 (6.0)
Hispanic	15 036 (7.8)
American Indian or Alaska Native	768 (0.4)
Other race and ethnicity^a^	4065 (2.1)
Pacific Islander	1975 (1.0)
White	138 702 (72.1)
Education	
Some high school or less	6389 (3.3)
High school graduate	38 214 (19.9)
Some college	48 474 (25.2)
College degree	68 213 (35.5)
Graduate degree	30 981 (16.1)
Employment status^b^	
Full-time	81 239 (42.3)
Gig or contract	1199 (0.6)
Homemaker	12 399 (6.4)
Part-time	20 759 (10.8)
Retired	31 167 (16.2)
Self-employed	11 330 (5.9)
Student	10 950 (5.7)
Unemployed	23 223 (12.1)
Annual household income category, $	
<25 000	43 566 (22.7)
25 000 to <50 000	47 721 (24.8)
50 000 to <75 000	34 861 (18.1)
75 000 to <100 000	25 692 (13.4)
≥100 000	40 431 (21.0)
Urbanicity	
1 (Most urban)	46 969 (24.4)
2	41 572 (21.6)
3	47 452 (24.7)
4	24 726 (12.9)
5	21 863 (11.4)
6 (Most rural)	9689 (5.0)
Additional household residents	
0	25 403 (13.2)
1	54 885 (28.5)
2	41 666 (21.7)
3 or More	70 317 (36.6)
Positive COVID-19 test	17 425 (9.1)
PHQ-9 score, mean (SD)	7.2 (6.8)
Proportion of population at home, mean (SD)	0.2 (0)

Abbreviations: PHQ-9, Patient Health Questionnaire-9.

^a^ Other refers to individuals who selected other when presented with a list of race and ethnicity categories.

^b^ Employment missing for n = 5.

**Table 2.** Additional Participant-Level Features Used in Regression Models^a^

Covariates	Mean (SD) (N = 192 271)
Month of pandemic (5 = May 2020)	14.0 (6.7)
COVID-19 cases in county, 14 d^b^	23.3 (29.7)
COVID-19 cases in state, 14 d	23.4 (27.0)
COVID-19 deaths in county, 14 d	0.35 (0.51)
COVID-19 deaths in state, 14 d	0.35 (0.38)
Masks required in public, 14 d^c^	0.5 (0.5)
Some required school closures, 14 d	0.7 (0.4)
Recommended workplace changes, 14 d	0.7 (0.4)
Recommended stay at home, 14 d	0.6 (0.5)
Reduced public transport, 14 d	0.4 (0.5)
Restrictions on large gatherings, 14 d	0.7 (0.5)
Recommended public event cancellation, 14 d	0.8 (0.4)
Precipitation, 14 d, mm/10	24.8 (80.3)
Maximum temperature (degrees Fahrenheit), 14 d	68.5 (17.5)
Minimum temperature (degrees Fahrenheit), 14 d	44.3 (3.1)

^a^ Missing observations: COVID-19 cases in county, n = 9546; COVID-19 cases in state, n = 9484; COVID-19 deaths in county, n = 9546; COVID-19 deaths in state, n = 9484; weather data, n = 6609.

^b^ Cases and deaths are reported as proportion of population, multiplied by 1000.

^c^ Policies are reported as proportion of past 14 d with policy present, ie, 0.5 means on average 50% of past 14 d.

In mixed-effects linear regression models, a greater mean county-level proportion of individuals not leaving home was associated with a greater level of depression symptoms (unadjusted β, 2.68; 95% CI, 1.65-3.72; adjusted β, 2.58; 95% CI, 1.57-3.58), meaning a 10% increase in the proportion staying at home was associated with a 0.27-point increase in PHQ-9, on average. The Figure illustrates the effect-size estimates in models incorporating county and state COVID-19 activity, inclusion of COVID-19 pandemic restrictions, and weather. Full model results are reported in Table 3. Some COVID-19 pandemic restrictions demonstrated significant independent associations with depressive symptom severity, including mandatory mask-wearing in public (β, 0.23; 95% CI, 0.15-0.30 and policies cancelling public events (β, 0.37; 95% CI, 0.22-0.51); estimated effects of restrictions were similar when staying at home was omitted from regression models (eTable 1 in Supplement 1).

**Figure.**
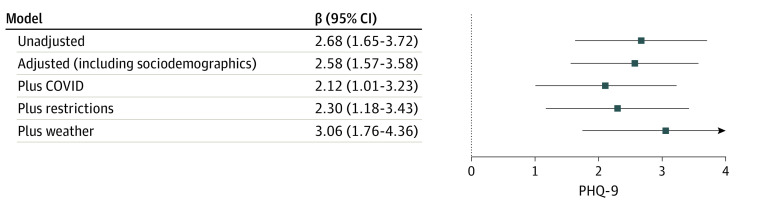
Forest Plot of Point Estimates of Coefficients for Lack of Mobility in Regression Models of Depression Severity

**Table 3.** Linear Mixed-Effects Regression Models of Depression Severity

Features	Demographic model (192 266 observations)		COVID-19 cases and deaths (182 722 observations)		COVID-19 restrictions (182 722 observations)		Weather (179 479 observations)	
	Estimates (95% CI)	*P* value	Estimates (95% CI)	*P* value	Estimates (95% CI)	*P* value	Estimates (95% CI)	*P* value
Proportion not leaving home^a^	2.58 (1.57 to 3.58)	<.001	2.12 (1.01 to 3.23)	<.001	2.30 (1.18 to 3.43)	<.001	3.06 (1.76 to 4.36)	<.001
COVID-19 cases, by county	NA	NA	−0.01 (−0.03 to 0.02)	.69	0 (−0.03 to 0.02)	.77	0 (−0.03 to 0.02)	.72
COVID-19 cases, by state	NA	NA	0.03 (0 to 0.06)	.04	0.02 (0 to 0.05)	.09	0.03 (0 to 0.05)	.06
COVID-19 deaths, by county	NA	NA	0.36 (−0.49 to 1.21)	.41	0.29 (−0.56 to 1.14)	.51	0.23 (−0.62 to 1.08)	.59
COVID-19 deaths, by state	NA	NA	−0.90 (−2.14 to 0.33)	.15	−1.20 (−2.43 to 0.04)	.06	−0.86 (−2.11 to 0.39)	.18
Masks required in public	NA	NA	NA	NA	0.23 (0.15 to 0.30)	<.001	0.25 (0.17 to 0.33)	<.001
Some required school closures	NA	NA	NA	NA	−0.11 (−0.20 to −0.02)	.02	−0.10 (−0.19 to −0.01)	.03
Recommended workplace changes	NA	NA	NA	NA	−0.07 (−0.19 to 0.05)	.25	−0.07 (−0.19 to 0.05)	.27
Recommended stay at home	NA	NA	NA	NA	0.06 (−0.03 to 0.16)	.18	0.07 (−0.02 to 0.16)	.13
Reduced public transport	NA	NA	NA	NA	0.04 (−0.06 to 0.13)	.45	0.05 (−0.04 to 0.15)	.27
Restrictions on large gatherings	NA	NA	NA	NA	−0.12 (−0.25 to 0)	.06	−0.10 (−0.22 to 0.03)	.14
Recommended public event cancellation	NA	NA	NA	NA	0.37 (0.22 to 0.51)	**<.001**	0.39 (0.24 to 0.54)	**<.001**
Precipitation, tenths of mm	NA	NA	NA	NA	NA	NA	0	.13
Maximum temperature, degrees F	NA	NA	NA	NA	NA	NA	0 (−0.01 to 0)	.51
Minimum temperature, degrees F	NA	NA	NA	NA	NA	NA	0.03 (−0.01 to 0.07)	.11

Abbreviation: NA, not applicable.

^a^ Features reflecting county-level averaged over past 14 d: proportion not leaving home; COVID-19 cases and deaths by county (proportion of population, x100), precipitation, and temperature. Policy measures reflect presence of state-level restrictions over past 14 days.

In sensitivity analysis, we examined the effect of considering 1 randomly selected observation per respondent, rather than the index observation; results were not meaningfully different, with β coefficient in the sociodemographic model of 2.58 (95% CI, 1.57-3.58; *P* < .001). Additional models can be found in (eTable 2 in Supplement 1. Results were also similar when all observations (249 038 observations for the 192 266 respondents) were included, with clustered standard errors ( β, 2.44; 95% CI, 1.75-31.4; *P* < .001). We further considered whether the magnitude of association between mobility and depressive symptoms changed after widespread availability of vaccination, extending the basic sociodemographic model to include an interaction between mobility and survey completion before or on/after May 1, 2021, the date when vaccination became available to all adults in the US.^31^ Because this term was significant (*t* = −2.11; *P* = .03), models were refit in the cohort before (n = 129 852) and on or after May 1, 2021 (n = 62 414). The magnitude of model coefficient was similar in both the prevaccine period (2.68; 95% CI, 1.45-3.90; *P* < .001) and the postvaccine period (2.49; 95% CI, 0.14-4.85; *P* = .04). The Video illustrates the association between the proportion staying at home and mean PHQ-9, by state, over time. State labels are colored to indicate COVID-19 deaths over the prior 2 weeks. Notably, the associations are apparent in some, but not all, time periods.

**Video.** Association Between State-Level Proportion of Individuals Staying at Home and Mean Patient Health Questionnaire-9 Score Over Time State labels are colored to indicate COVID-19 deaths over the prior 2 weeks as a proportion of total state population. Notably, the associations are apparent in some, but not all, time periods.

Because other county-level features could confound associations between mobility and depressive symptoms, we then fit additional regression models incorporating additional county population features that could associate with mobility (eTable 3 in Supplement 1 and Table 3). Incorporating population density, employment characteristics, and poverty measures did not meaningfully change the association between community mobility and mood (eTable 4 and eFigure in Supplement 1). Likewise, adding a term for political affiliation (considered categorically) to regression models did not meaningfully change the association between mobility and depressive symptoms (in sociodemographic-adjusted models: β,  2.59; 95% CI, 1.59-3.60).

To further address the risk for confounding, we considered the change in the proportion of adults in a community staying at home compared with 30 days prior and reasoned that any unmeasured county-level confounding effect sizes would be unlikely to change in this time frame. We found a significant association between an increase in the proportion of staying at home and greater depressive symptoms (unadjusted β,  3.34; 95% CI, 2.15-4.53; adjusted β,  2.25; 95% CI, 1.07-3.43) (eTable 5 in Supplement 1).

Finally, we drew on the subset of survey participants (n = 31 158) who returned for at least 1 subsequent wave and examined the association between change in community mobility between the 2 waves and change in depressive severity from the index to the next survey. Characteristics of this returning population, compared with those who did not return, are summarized in the eTable 6 in Supplement 1. This group was on average statistically significantly older, less likely to be employed full time, and had lower depression severity at initial visit. In this subgroup, an increase in the proportion of community staying at home was significantly associated with greater individual depression severity (unadjusted β,  3.04; 95% CI, 1.45-4.62; adjusted β,  3.07; 95% CI, 1.44-4.71).

## Discussion

This large-scale survey study found evidence of an association between community mobility—specifically, the proportion of individuals who did not leave home in a given time and place—and greater levels of depressive symptoms. These associations did not appear to be fully explainable by county stringency policies per se, nor by other factors, including weather that might diminish mobility. Our results cannot directly address a causal relationship between restrictions associated with COVID-19 and depressive symptoms. However, they do suggest that a potential correlate of reduced mobility during COVID-19 was greater risk for depressive symptoms.

We conducted multiple additional analyses to address the risk of confounding at the county level. First, we examined population density, employment, and poverty; including these terms did not fully explain the observed associations. Second, we considered a change in the proportion staying at home from 30 days prior, as socioeconomic county-level features are unlikely to change on this scale. Third, in a subset of participants who returned for a subsequent survey, we examined whether their change in depressive symptoms was associated with a change in community mobility. In all of these analyses, results were consistent with the primary analysis in supporting an association.

We elected to use community measures, rather than individual measures, for 2 reasons. First, we sought to understand community-level effects—ie, whether living in such a community matters, rather than individual mobility per se. Second, the cross-sectional nature of the survey would complicate interpretation of such an analysis if individual behavior was examined. For example, depressive symptoms could cause a reduction in mobility, arise from a reduction in mobility, or neither of these.

We identified statistically significant, but very modest, associations between some COVID-19 containment policies and depressive symptoms. In particular, the requirement of public mask-wearing and cancellation of public events were associated with fractional increases in depressive symptoms in models adjusted for sociodemographic features. The persistence of these outcomes in models adjusted for county-level COVID-19 acuity, as well as month of the COVID-19 pandemic, suggests they are not merely proxies for local severity of the pandemic. Conversely, while they suggest the possibility that such policies could impact mood, they also indicate that any such impact is likely to be extremely modest, particularly when weighed against other public health benefits, as a recent large-scale study indicates.^32^ Any association of these policies was substantially smaller than the magnitude of association with community mobility.

Our results are consistent with a prior study investigating the relationship between regional mobility and Google searches for mental health topics early in the COVID-19 pandemic.^33^ At an individual level, a study of 682 young adults using wearable sensors found that diminished activity in the initial COVID-19 pandemic period was associated with greater risk for depressive symptoms.^34^ A similar study^35^ using wearable sensors in 10 older adults likewise found that diminished mobility was associated with an increase in depressive symptoms among older adults. A limitation in these individual-level studies is the risk of reverse causation—ie, that reduced individual mobility may reflect onset of depressive symptoms, rather than contributing to them.

### Limitations

This study has limitations. First, it relies on cross-sectional associations, limiting our ability to apply causal inference. We cannot determine whether the reductions in mobility are responsible for the depressive symptoms we observe; however, at minimum, can exclude reverse causation as an explanation.

Second, while we examined multiple potential confounding associations, we cannot exclude the possibility of unobserved confounding. For example, some other factor, such as economic deprivation, could contribute to both lower community mobility and greater depressive symptoms. On the other hand, including multiple sociodemographic and socioeconomic features in regression models does not meaningfully change the associations we observe, and it seems likely that these features could at least serve as proxies for many of the potential confounders that could otherwise explain this association.

Third, the extent to which results would generalize from this population of individuals in the United States willing to complete internet-based surveys remains to be determined. In prior work, we have demonstrated that our survey results are generally associated with administrative measures^36^ and with more expensive probability-based sampling^37^ methods that are not predicated on internet access. Similarly, the social media mobility measures represent only a small subset of any given county. We note that the US Census American Community Survey^38^ indicates that almost all adults have a cell phone and 85% of adults have a smartphone, even if they are among the quarter of US households that do not have internet at home. Still, understanding the contexts in which nonprobability sampling is most comparable with traditional, far more costly probability designs remains an evolving area of work.^17,20^ Here, as we maintained sampling scheme and frame over time, our results are unlikely to be impacted by many of the typical challenges to validity with this design.^17,20^

## Conclusions

In this survey study, we found that depressive symptoms during the COVID-19 pandemic were associated with an element of local environment, namely the extent to which people in a community leave home. Neither COVID-19–related restrictions nor recent COVID-19 activity explained this association. While we cannot determine causation, the potential importance of interventions aimed at increasing social engagement during times of limited mobility merits consideration for future pandemics or other long-lasting disasters.

## Notes

Supplement 1. **eFigure.** Estimates of Association Between Lack of Mobility and Depression Severity, Adjusted for Additional County Characteristics **eTable 1.** Linear Mixed-Effects Models Including COVID-19 Restriction Policies but Omitting Proportion of Individuals Staying at Home **eTable 2.** Linear Mixed-Effects Models Using Randomly Selected Survey Rather Than First Survey **eTable 3.** County Characteristics Used in Supplemental Analyses **eTable 4.** Linear Mixed-Effects Models for Depression Severity, Adjusting for County Characteristics **eTable 5.** Linear Mixed-Effects Models for Depression Severity, Based on 30 Day Change in Proportion of Individuals Staying at Home **eTable 6.** Characteristics of Returning Survey Respondents **eAppendix.** Survey Questions Used in Regression Analyses

Supplement 2.
Data Sharing Statement
