# Supplementary material for: Community Mobility and Depressive Symptoms During the COVID-19 Pandemic in the United States
Source: JAMA Netw Open. 2023 Sep 27;6(9):e2334945. doi: 10.1001/jamanetworkopen.2023.34945 (PMC10534266; doi:10.1001/jamanetworkopen.2023.34945)
Supplement: Supplement 1. — eFigure. Estimates of Association Between Lack of Mobility and Depression Severity, Adjusted for Additional County Characteristics eTable 1. Linear Mixed-Effects Models Including COVID-19 Restriction Policies but Omitting Proportion of Individuals Staying at Home eTable 2. Linear Mixed-Effects Models Using Randomly Selected Survey Rather Than First Survey eTable 3. County Characteristics Used in Supplemental Analyses eTable 4. Linear Mixed-Effects Models for Depression Severity, Adjusting for County Characteristics eTable 5. Linear Mixed-Effects Models for Depression Severity, Based on 30 Day Change in Proportion of Individuals Staying at Home eTable 6. Characteristics of Returning Survey Respondents eAppendix. Survey Questions Used in Regression Analyses [file jamanetwopen-e2334945-s001.pdf]

Supplemental Online Content

Perlis RH, Lunz Trujillo K, Safarpour A, et al. Community mobility and depressive symptoms during the COVID-19 pandemic in the United States. *JAMA Netw Open*. 2023;6(9):e2334945. doi:10.1001/jamanetworkopen.2023.34945

eFigure. Estimates of Association Between Lack of Mobility and Depression Severity, Adjusted for Additional County Characteristics

eTable 1. Linear Mixed Effects Models Including COVID-19 Restriction Policies but Omitting Proportion of Individuals Staying at Home

eTable 2. Linear Mixed Effects Models Using Randomly-Selected Survey Rather Than First Survey

eTable 3. County Characteristics Used in Supplemental Analyses

eTable 4. Linear Mixed Effects Models for Depression Severity, Adjusting for County Characteristics

eTable 5. Linear Mixed-Effects Models for Depression Severity, Based on 30 Day Change in Proportion of Individuals Staying at Home

eTable 6. Characteristics of Returning Survey Respondents

eAppendix. Survey Questions Used in Regression Analyses

This supplemental material has been provided by the authors to give readers additional information about their work.

© 2023 Perlis R et al. *JAMA Network Open.*

eTable 1. Linear mixed effects models including COVID-19 restriction policies but omitting proportion of individuals staying at home

	**Restrictions only**			Plus sociodemographics			Plus COVID acuity		
Features	*Estimates*	*CI*	*p*	*Estimates*	*CI*	*p*	*Estimates*	*CI*	*p*
**Masks required in public**	0.24	0.16 – 0.32	**<0.001**	0.24	0.16 – 0.31	**<0.001**	0.22	0.14 – 0.29	**<0.001**
**Some required school closures**	-0.10	-0.20 – -0.01	**0.026**	-0.09	-0.18 – -0.00	**0.045**	-0.09	-0.18 – -0.00	**0.044**
**Recommended workplace changes**	-0.05	-0.18 – 0.07	0.411	-0.06	-0.18 – 0.06	0.326	-0.07	-0.20 – 0.05	0.228
**Recommended stay at home**	0.06	-0.03 – 0.15	0.166	0.10	0.01 – 0.19	**0.023**	0.09	-0.00 – 0.18	0.057
**Reduced public transport**	-0.02	-0.12 – 0.07	0.629	0.04	-0.06 – 0.13	0.443	0.05	-0.04 – 0.14	0.299
**Restrictions on large gatherings**	-0.06	-0.19 – 0.06	0.324	-0.09	-0.21 – 0.03	0.149	-0.11	-0.24 – 0.02	0.089
**Recommended public event cancellation**	0.20	0.06 – 0.35	**0.007**	0.35	0.20 – 0.49	**<0.001**	0.36	0.21 – 0.50	**<0.001**
**Observations**	192271			192266			182722		

eTable 2. Linear mixed effects models using randomly-selected survey rather than first survey

	**Demographic model**			Plus COVID cases and deaths			Plus COVID restrictions			Plus Weather		
Features	*Estimates*	*CI*	*p*	*Estimates*	*CI*	*p*	*Estimates*	*CI*	*p*	*Estimates*	*CI*	*p*
**Proportion not leaving home**	2.58	1.57 – 3.58	**<0.001**	2.12	1.01 – 3.23	**<0.001**	2.30	1.18 – 3.43	**<0.001**	3.06	1.76 – 4.36	**<0.001**
**COVID cases, by county**				-0.01	-0.03 – 0.02	0.690	-0.00	-0.03 – 0.02	0.768	-0.00	-0.03 – 0.02	0.724
**COVID cases, by state**				0.03	0.00 – 0.06	**0.039**	0.02	-0.00 – 0.05	0.091	0.03	-0.00 – 0.05	0.062
**COVID deaths, by county**				0.36	-0.49 – 1.21	0.405	0.29	-0.56 – 1.14	0.505	0.23	-0.62 – 1.08	0.593
**COVID deaths, by state**				-0.90	-2.14 – 0.33	0.152	-1.20	-2.43 – 0.04	0.058	-0.86	-2.11 – 0.39	0.178
**Masks required in public**							0.23	0.15 – 0.30	**<0.001**	0.25	0.17 – 0.33	**<0.001**
**Some required school closures**							-0.11	-0.20 – -0.02	**0.020**	-0.10	-0.19 – -0.01	**0.027**
**Recommended workplace changes**							-0.07	-0.19 – 0.05	0.237	-0.07	-0.19 – 0.05	0.269
**Recommended stay at home**							0.06	-0.03 – 0.16	0.184	0.07	-0.02 – 0.16	0.127
**Reduced public transport**							0.04	-0.06 – 0.13	0.438	0.05	-0.04 – 0.15	0.266
**Restrictions on large gatherings**							-0.12	-0.25 – 0.00	0.059	-0.10	-0.22 – 0.03	0.136
**Recommended public event cancellation**							0.37	0.22 – 0.51	**<0.001**	0.39	0.24 – 0.54	**<0.001**
**Precipitation (tenths of mm)**										0.00	-0.00 – 0.00	0.133
**Maximum temperature (degrees F)**										-0.00	-0.01 – 0.00	0.512
**Minimum temperature (degrees F)**										0.03	-0.01 – 0.07	0.114
**Observations**	192266			182722			182722			179479		

© 2023 Perlis R et al. *JAMA Network Open.*

eTable 3. County characteristics employed in supplemental analyses

	Overall (N=192271)
**Population Density (per km)**	
Mean (SD)	2016.2 (7382.0)
**Unemployment Rate**	
Mean (SD)	7.7 (2.3)
**Percent Employed - Agriculture**	
Mean (SD)	1.2 (1.7)
**Percent Employed - Services**	
Mean (SD)	49.2 (5.5)
**Poverty Rate**	
Mean (SD)	12.6 (4.5)
**Deep Poverty Rate**	
Mean (SD)	5.8 (2.2)
**Average Household Size**	
Mean (SD)	2.6 (0.2)
**Percent Owning Home**	
Mean (SD)	65.2 (10.4)
**Household Income**	
Mean (SD)	70736.5 (18396.6)

© 2023 Perlis R et al. *JAMA Network Open.*

eTable 4. Linear mixed effects models for depression severity, adjusting for county characteristics

	**Sociodemographic**			Plus Population Density			Plus Employment			Plus Poverty		
Features	*Estimates*	*CI*	*p*	*Estimates*	*CI*	*p*	*Estimates*	*CI*	*p*	*Estimates*	*CI*	*p*
**Stay at home (%)**	2.58	1.57 – 3.58	**<0.001**	2.29	1.28 – 3.30	**<0.001**	2.18	1.15 – 3.20	**<0.001**	2.13	1.09 – 3.16	**<0.001**
**Population Density (per 1k km)**				0.01	0.01 – 0.02	**<0.001**	0.01	0.01 – 0.02	**<0.001**	0.01	0.00 – 0.02	**0.003**
**Unemployment Rate (%)**							0.14	-0.04 – 0.33	0.136	-0.00	-0.21 – 0.21	0.989
**Agriculture Employment (%)**							-0.44	-0.66 – -0.21	**<0.001**	-0.51	-0.74 – -0.28	**<0.001**
**Services Employment (%)**							-0.08	-0.15 – -0.01	**0.025**	-0.12	-0.21 – -0.03	**0.006**
**Poverty Rate (%)**										0.30	0.03 – 0.57	**0.027**
**Deep Poverty Rate (%)**										-0.12	-0.56 – 0.32	0.594
**Average Household Size (n)**										-0.01	-0.20 – 0.18	0.948
**Proportion Owning Home (%)**										-0.00	-0.06 – 0.06	0.933
**Median Household Income ($10k)**										0.02	-0.02 – 0.06	0.334
**Observations**	192266			192259			192259			192259		

© 2023 Perlis R et al. *JAMA Network Open.*

eFigure. Estimates of association between lack of mobility and depression severity, adjusted for additional county characteristics

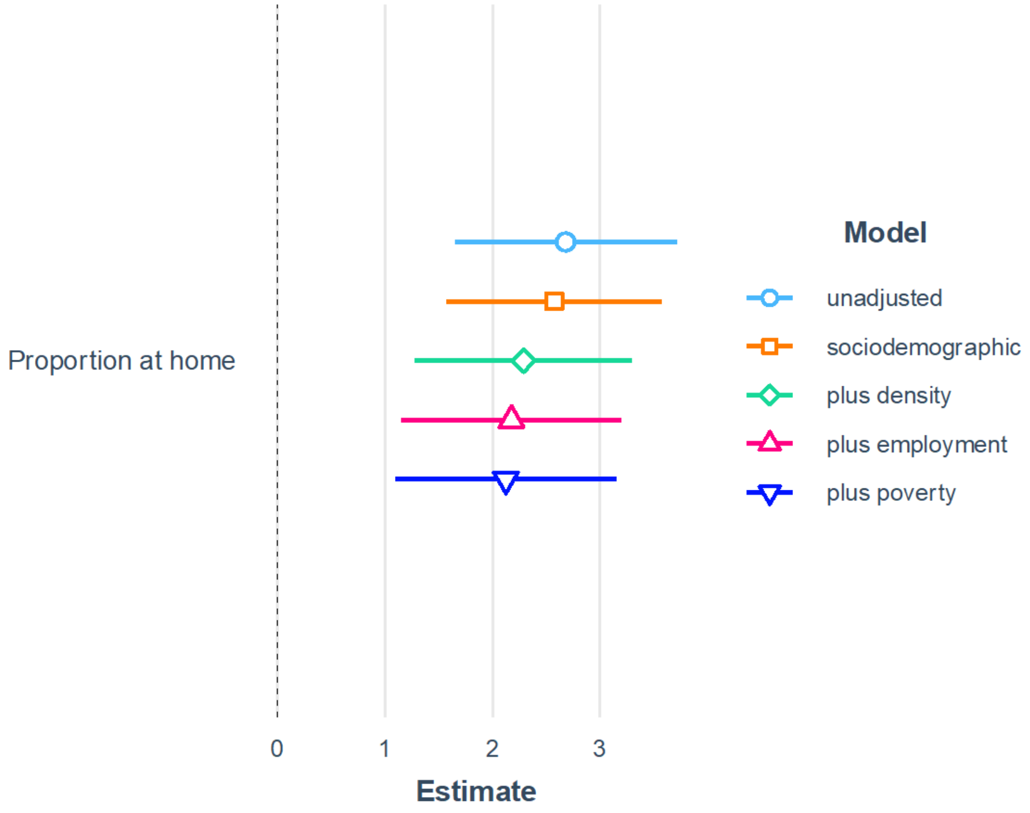

© 2023 Perlis R et al. *JAMA Network Open.*

eTable 5. Linear mixed effects models for depression severity, based on 30d change in proportion of individuals staying at home

	**Unadjusted model**			**Adjusted model**		
Predictors	*Estimates*	*CI*	*p*	*Estimates*	*CI*	*p*
30-day change in proportion at home	3.34	2.15 – 4.53	**<0.001**	2.25	1.07 – 3.43	**<0.001**
Pandemic month				-0.02	-0.02 – -0.01	**<0.001**
Respondent age (in years)				-0.11	-0.11 – -0.11	**<0.001**
**Education** (vs No High School Degree)						
High School Graduate				-0.82	-0.99 – -0.65	**<0.001**
Some College				-0.74	-0.91 – -0.57	**<0.001**
College Degree				-1.36	-1.53 – -1.19	**<0.001**
Graduate Degree				-0.79	-0.97 – -0.61	**<0.001**
**Household income (vs less than $25k/year)**						
$25k to less than $50k/year				-0.65	-0.74 – -0.57	**<0.001**
$50k to less than $75k/year				-1.34	-1.44 – -1.25	**<0.001**
$75k to less than $100k/year				-1.71	-1.82 – -1.61	**<0.001**
$100k/year or more				-1.69	-1.79 – -1.58	**<0.001**
**Current employment (vs Full-time)**						

© 2023 Perlis R et al. *JAMA Network Open.*

Gig/Contract	1.76	1.39 – 2.12	**<0.001**
Home-maker	-0.20	-0.33 – -0.07	**0.002**
Part-time	0.19	0.08 – 0.29	**<0.001**
Retired	0.16	0.05 – 0.27	**0.004**
Self-employed	0.26	0.13 – 0.38	**<0.001**
Student	0.70	0.56 – 0.84	**<0.001**
Unemployed	1.97	1.86 – 2.07	**<0.001**
**Race and ethnicity (vs Black)**			
Asian American	0.28	0.13 – 0.43	**<0.001**
Hispanic	0.75	0.61 – 0.89	**<0.001**
Native American	1.41	0.94 – 1.87	**<0.001**
Other	0.79	0.57 – 1.00	**<0.001**
Pacific Islander	1.34	1.04 – 1.64	**<0.001**
White	1.21	1.11 – 1.31	**<0.001**
Male Gender	-0.57	-0.63 – -0.51	**<0.001**
**Urbanicity (vs 1= most urban)**			
2	-0.20	-0.29 – -0.10	**<0.001**
3	-0.15	-0.24 – -0.05	**0.002**

© 2023 Perlis R et al. *JAMA Network Open.*

4	-0.23	-0.34 – -0.12	**<0.001**
5	-0.27	-0.39 – -0.16	**<0.001**
6 (Most rural)	-0.19	-0.34 – -0.04	**0.014**
**Total household residents (vs 1)**			
2 household residents	-0.14	-0.23 – -0.04	**0.006**
3 household residents	0.13	0.03 – 0.23	**0.014**
4 or more additional residents	0.42	0.32 – 0.52	**<0.001**
Positive COVID-19 test	1.26	1.16 – 1.37	**<0.001**

© 2023 Perlis R et al. *JAMA Network Open.*

eTable 6. Characteristics of returning survey respondents

	FALSE (N=161113)	TRUE (N=31158)	Total (N=192271)	p value
**Respondent age (in years)**				< 0.001
Mean (SD)	41.3 (16.0)	52.4 (16.0)	43.1 (16.5)	
**Gender**				0.222
Female	105807 (65.7%)	20574 (66.0%)	126381 (65.7%)	
Male	55306 (34.3%)	10584 (34.0%)	65890 (34.3%)	
**Race and ethnicity**				< 0.001
African American	17122 (10.6%)	3155 (10.1%)	20277 (10.5%)	
Asian American	9784 (6.1%)	1664 (5.3%)	11448 (6.0%)	
Hispanic	13441 (8.3%)	1595 (5.1%)	15036 (7.8%)	
Native American	694 (0.4%)	74 (0.2%)	768 (0.4%)	
Other	3628 (2.3%)	437 (1.4%)	4065 (2.1%)	
Pacific Islander	1753 (1.1%)	222 (0.7%)	1975 (1.0%)	
White	114691 (71.2%)	24011 (77.1%)	138702 (72.1%)	
**Education**				< 0.001
Some High School or Less	5819 (3.6%)	570 (1.8%)	6389 (3.3%)	
High School Graduate	32131 (19.9%)	6083 (19.5%)	38214 (19.9%)	
Some College	40709 (25.3%)	7765 (24.9%)	48474 (25.2%)	
College Degree	56322 (35.0%)	11891 (38.2%)	68213 (35.5%)	
Graduate Degree	26132 (16.2%)	4849 (15.6%)	30981 (16.1%)	
**Employment[a]**				< 0.001
Full-time	70668 (43.9%)	10571 (33.9%)	81239 (42.3%)	
Gig/Contract	1036 (0.6%)	163 (0.5%)	1199 (0.6%)	
Home-maker	10253 (6.4%)	2146 (6.9%)	12399 (6.4%)	
Part-time	17831 (11.1%)	2928 (9.4%)	20759 (10.8%)	
Retired	21563 (13.4%)	9604 (30.8%)	31167 (16.2%)	
Self-employed	9524 (5.9%)	1806 (5.8%)	11330 (5.9%)	
Student	10303 (6.4%)	647 (2.1%)	10950 (5.7%)	
Unemployed	19930 (12.4%)	3293 (10.6%)	23223 (12.1%)	
**Income category**				< 0.001
Less than $25k/year	37070 (23.0%)	6496 (20.8%)	43566 (22.7%)	

© 2023 Perlis R et al. *JAMA Network Open.*

	FALSE (N=161113)	TRUE (N=31158)	Total (N=192271)	p value
$25k to less than $50k/year	39301 (24.4%)	8420 (27.0%)	47721 (24.8%)	
$50k to less than $75k/year	28667 (17.8%)	6194 (19.9%)	34861 (18.1%)	
$75k to less than $100k/year	21435 (13.3%)	4257 (13.7%)	25692 (13.4%)	
$100k/year or more	34640 (21.5%)	5791 (18.6%)	40431 (21.0%)	
**Urbanicity**				< 0.001
Most urban	40046 (24.9%)	6923 (22.2%)	46969 (24.4%)	
2	34744 (21.6%)	6828 (21.9%)	41572 (21.6%)	
3	39576 (24.6%)	7876 (25.3%)	47452 (24.7%)	
4	20546 (12.8%)	4180 (13.4%)	24726 (12.9%)	
5	18153 (11.3%)	3710 (11.9%)	21863 (11.4%)	
Most rural	8048 (5.0%)	1641 (5.3%)	9689 (5.0%)	
**Additional household members**				< 0.001
0	20755 (12.9%)	4648 (14.9%)	25403 (13.2%)	
1	44194 (27.4%)	10691 (34.3%)	54885 (28.5%)	
2	34408 (21.4%)	7258 (23.3%)	41666 (21.7%)	
3+	61756 (38.3%)	8561 (27.5%)	70317 (36.6%)	
**Positive COVID test**				< 0.001
No	144885 (89.9%)	29961 (96.2%)	174846 (90.9%)	
Yes	16228 (10.1%)	1197 (3.8%)	17425 (9.1%)	
**PHQ-9 sum**				< 0.001
Mean (SD)	7.5 (6.9)	5.6 (6.2)	7.2 (6.8)	

a. employment status missing for 5 individuals who did not return

© 2023 Perlis R et al. *JAMA Network Open.*

eAppendix. Survey questions used in regression analyses.

[house] Apart from you, how many other people currently live in your household? -
[house_0_6] Children under age 6:
[house_6_12] Children age 6 to 12:
[house_13_17] Children age 13 to 17:
[house_18_59] Adults age 18 to 59 (other than you):
[house_60] Adults over the age of 60 (other than you) :

[covid] Have you ever been diagnosed with coronavirus (COVID-19)?
1 = Yes, I was diagnosed by a medical professional
2 = No, I was not diagnosed but I think I may have it now
3 = No, I was not diagnosed but I think I had it previously and recovered
4 = No, I was not diagnosed and I do not think I ever had it
5 = I am not sure

[cov_test] Have you been tested for coronavirus (COVID-19)?
1 = Yes, and I tested positive for COVID-19 at least once
2 = Yes, and I tested negative for COVID-19 every time
3 = No, I wanted to but was not able to get a test
4 = No, I never tried to get tested

[test_home] Have you ever used an "at-home" or "rapid" test for COVID-19? That would be a test that did not require you to go to a doctor's office or send samples to a testing facility.
1 = No
2 = Yes, and I always tested negative

© 2023 Perlis R et al. *JAMA Network Open.*

[employ] Which of the following best describes your current employment status?
Full-time = Full-time
Part-time = Part-time
Self-employed = Self-employed
Unemployed = Unemployed
Home-maker = Home-maker
Student = Student
Retired = Retired
Gig/Contract = Gig/Contract

[phq9] Over the last two weeks, how often have you been bothered by the following problems? -
[phq9_1] Little interest or pleasure in doing things
[phq9_2] Feeling down, depressed, or hopeless
[phq9_3] Trouble falling or staying asleep, or sleeping too much
[phq9_4] Feeling tired or having little energy
[phq9_5] Poor appetite or overeating
[phq9_6] Feeling bad about yourself - or that you are a failure or have let yourself or your family down
[phq9_7] Trouble concentrating on things, such as reading the newspaper or watching television
[phq9_8] Moving or speaking so slowly that other people could have noticed -- or so fidgety or restless that you have been moving a lot more than usual
[phq9_9] Thoughts that you would be better off dead, or thoughts of hurting yourself in some way

1 = Not at all
2 = Several days
3 = More than half the days
4 = Nearly every day

© 2023 Perlis R et al. *JAMA Network Open.*

[race] Race as provided by vendor (select one)
African American = African American
Asian American = Asian American
Hispanic = Hispanic
Native American = Native American
Pacific Islander = Pacific Islander
White = White
Other = Other

[age] Respondent age (in years)
-- Numeric value

[education] Education level (numerical)
1 = Some High School or Less
2 = High School Graduate
3 = Some College
4 = College Degree
5 = Graduate Degree

[income_cat_10] What was the total combined income of your household for the past year?
1 = Under 10K
2 = 10k to under 15k
3 = 15K to under 25K
4 = 25K to under 35K
5 = 35K to under 50K
6 = 50K to under 75K
7 = 75K to under 100K

© 2023 Perlis R et al. *JAMA Network Open.*

8 = 100K to under 150K
9 = 150K to under 200K
10 = 200K and over

[region] US Region
Northeast = Northeast
Midwest = Midwest
South = South
West = West

[fips] State-County FIPS Code
-- Numeric value

[urbanicity] NCHS Urban-Rural Classification 2013
1 = Large central metro
2 = Large fringe metro
3 = Medium metro
4 = Small metro
5 = Micropolitan
6 = Non-Core

[urban_type] Urban type (NCHS)
Urban = Urban
Suburban = Suburban
Rural = Rural

© 2023 Perlis R et al. *JAMA Network Open.*
