# Supplementary material for: Community Mobility and Depressive Symptoms During the COVID-19 Pandemic in the United States
Source: JAMA Netw Open. 2023 Sep 27;6(9):e2334945. doi: 10.1001/jamanetworkopen.2023.34945 (PMC10534266; doi:10.1001/jamanetworkopen.2023.34945)
Supplement: Supplement 2. — Data Sharing Statement [file jamanetwopen-e2334945-s002.pdf]

Data Sharing Statement

Perlis. Community Mobility and Depressive Symptoms During the COVID-19 Pandemic in the United States. *JAMA Netw Open*. Published September 27, 2023.
doi:10.1001/jamanetworkopen.2023.34945

Data
Data available: Yes
Data types: Deidentified participant data
How to access data: covidstates.or
When available: beginning date: 06-01-2024

Supporting Documents
Document types: None

Additional Information
Who can access the data: approved data requests
Types of analyses: for any purpose
Mechanisms of data availability: via request to covidstates.org
